# Development and identification of a prognostic nomogram model for patients with mixed cell adenocarcinoma of the ovary

**DOI:** 10.1186/s13048-021-00896-9

**Published:** 2021-10-21

**Authors:** Huijie Wu, Shaotao Jiang, Peiwen Zhong, Weiru Li, Siyou Zhang

**Affiliations:** 1 Department of Gynecology, The First People’s Hospital of Foshan, Foshan, 528000 Guangdong China; 2 Department of HBP SURGERY II, Guangzhou First People’s Hospital, School of Medicine, South China University of Technology, Guangzhou, 510180 Guangdong China

**Keywords:** Mixed cell ovarian adenocarcinoma, Nomogram, SEER, Propensity score matching

## Abstract

**Background:**

Mixed cell ovarian adenocarcinoma (MCOA) is a malignant gynecologic tumor consisting of serous, mucous, and papillary tumor cells. However, the clinical features and prognosis of MCOA patients are unclear.

**Methods:**

In this study, univariate and multivariate Cox proportional risk models were performed to identify independent prognostic factors. The Kaplan–Meier method was used to assess the relationship between clinical characteristics and patient survival. Finally, a nomogram was constructed and validated to predict patient survival time, and the C-index was used to evaluate the efficacy of the nomogram.

**Results:**

A total of 2,818 patients diagnosed with MCOA were identified, and the 5-year survival rate was 62%. Univariate and multivariate Cox models suggested that age (HR=1.28, 95% CI[1.15,1.44]), grade (HR=1.26, 95% CI[1.12,1.41]), SEER stage (HR=1.63, 95% CI[1.25,2.13]) and AJCC (American Joint Committee on Cancer) stage (HR=1.59, 95% CI[1.36,1.86]) were independent prognostic factors for MCOA patients. After propensity score matching for age, grade, SEER stage, and AJCC stage, the 5-year survival rate was 69.7% for ovarian serous cystadenocarcinoma and 62.9% for ovarian papillary serous cystadenocarcinoma. These results mean that serous adenocarcinoma had the best prognosis of the three pathologic types of ovarian carcinoma (*p*<0.0001), with no significant difference between papillary serous cystadenocarcinoma and MCOA (*p*=0.712). Finally, a nomogram consisting of age, grade, SEER stage, and AJCC stage was established and validated to predict the survival time, with C-indices of 0.743 and 0.731, respectively.

**Conclusions:**

In summary, MCOA is uncommon, and age, grade, SEER stage, and AJCC stage are independent prognostic factors. Compared with other common malignant ovarian tumors, MCOA has a poor prognosis.

## Introduction

Ovarian cancer is one of the three major malignant tumors in gynecology, and its incidence is second only to cervical cancer and endometrial cancer. Because of the lack of effective screening methods for ovarian cancer, 75% of patients are found to be advanced. Female germ cell tumors are among the deadliest. Globally, 239,000 new cases (3.6% of all cancer cases) and 152,000 deaths (4.3% of all cancer deaths) are recorded each year [1]. To reduce mortality, awareness of ovarian cancer must be raised, including special types of ovarian cancer. The World Health Organization (WHO) divides epithelial ovarian cancer into several morphological categories based on cell type: serous ovarian carcinoma (SOC), mucinous ovarian carcinoma (MOC), endometrioid carcinoma (EC), ovarian clear cell carcinoma (OCCC), mixed cell ovarian adenocarcinoma (MCOA) and undifferentiated transitional cell Brenner tumor [2]. Among these types of epithelial ovarian cancer, the clinical characteristics and prognosis of MCOA patients remain unclear.

MCOA is a rare ovarian cancer in which the tumor is mixed with several types of malignant cells (such as serous, mucinous, and papillary cancer cells) [3, 4]. Mixed cell carcinoma is diagnosed when the proportion of malignant cells in the second component of ovarian tumors is greater than 10% [3, 5]. At present, no studies with large sample data have described this type of gynecologic malignancy. Previous studies of ovarian cancer mixed cell types have mostly included a few cases or case reports. A study containing 23 cases of mixed ovarian cancer showed that MCOA was clinically similar to high-grade serous ovarian carcinoma (HGSOC) [6]. In addition, SOC is one of the common types of epithelial ovarian cancer. SOC is < 5% low-grade serous ovarian carcinoma (LGSOC) [7] and mostly HGSOC, with a 10-year survival rate of 55% in early-stage patients [8].

Therefore, in this study, we screened SEER database patients diagnosed with MCOA to identify potential prognostic risk factors. Then, a nomogram was constructed to predict prognosis and assist clinical decision-making. In addition, survival analysis for serous adenocarcinoma and papillary serous cystadenocarcinoma patients was compared with that of MOCA patients after 1:1 propensity score matching (PSM). To the best of our knowledge, our study is a retrospective study with the largest sample size of MCOA.

## Materials and methods

### Patients

This study screened the SEER database of patients diagnosed with MCOA from 1975 to 2016. Patients with ovarian carcinomas coded 8323/3 mixed cell adenocarcinoma, 8441/3 serous cystadenocarcinoma NOS, and 8460/3 papillary serous cystadenocarcinoma according to the International Classification of Diseases for Oncology (ICD-O-3) rules were screened from the SEER database. Patients with an uncertain survival time or cancer special status were excluded. All patient data were downloaded through SEER*Stat software. Since the SEER database is a public database, the analysis of data from patients with ovarian mixed cell adenocarcinoma does not require informed consent and ethical review.

### Statistical analysis

 In this study, continuous variables (such as age and tumor size) were converted into categorical variables by X-tile [9] software with the optimal cutoff value through the enumeration method. Then, univariate and multivariate Cox proportional risk models were performed to identify independent prognostic factors for MCOA. The Kaplan–Meier method was used to assess the relationship between clinical characteristics and patient survival. Finally, the patients were randomly divided into two groups to construct and validate a nomogram. By integrating multiple predictors and drawing multiple lines with scales in proportion, the nomogram can easily calculate the risk of disease or the probability of survival of an individual, and the C-index was used to evaluate the efficacy of the nomogram. The nomogram was built and validated in R software using the *survival package* and the *rms package*. In addition, the prognosis of patients with different pathological types was compared, and PSM analysis of clinical characteristics was performed to reduce confounding bias.

## Results

### Basic clinical characteristics of mixed cell adenocarcinoma patients

A total of 2,818 MCOAs were included in this study from the SEER database of 1975 to 2016. The clinical characteristics of these patients are shown in Table 1. X-tile software was used to calculate the optimal cutoff values for age and tumor size based on survival time, and the 2,818 patients were divided into three groups. More than half (56.3%) of the patients were between 18-59 years old, 28.7% were in the 60-72 group, and 15% were in the 73-96 group. Patients were divided into three groups according to tumor size: 0-83 mm (*n *= 816, 24.7%), 84-155 mm (*n *= 1013, 30.6%), and 156-790 mm (*n *= 509, 15.4%). In addition, 38.2% of patients were in a poorly differentiated group, higher than well differentiated (8.4%), moderately differentiated (16.7%), and undifferentiated (21.8%). There were 662 (20%), 1059 (32%), and 1561 (47.2%) patients with localized, regional, and distant SEER stages, respectively. According to the AJCC staging system, stage III had the most people (*n* = 1003, 30.3%), followed by stage I (*n* = 960, 2%), stage II (*n* = 396, 12%), and stage IV (*n* = 392, 11.8%). In these mixed cell adenocarcinomas, 36% were bilateral (single primary), 30.9% were on the left ovary, and 30.3% were on the right ovary. Surgical treatment was performed in 95.9% of patients, with cell reduction as the main treatment (*n *= 1,281, 38.7%).

**Table 1:** Baseline characteristics of patients with mixed cell adenocarcinoma

Variable	Classification	Number (*n*=2,818)	Percentage (100%)
Race	White	2376	84.3
	Black	128	4.5
	Others	302	10.7
	Unknown	12	0.4
Age	18-59	1627	57.7
	60-72	789	28
	73-96	402	14.3
Size	0-83	668	23.7
	84-155	866	30.7
	156+	456	16.2
	Unknown	828	29.4
Grade	Well differentiated	225	8
	Moderately differentiated	461	16.4
	Poorly differentiated	1090	38.7
	Undifferentiated	623	22.1
	Unknown	419	14.9
SEER stage	Localized	551	19.6
	Regional	900	31.9
	Distant	1342	47.6
	Unknown	25	0.9
AJCC stage	Stage I	808	28.7
	Stage II	338	12
	Stage III	865	30.7
	Stage IV	331	11.7
	Unknown	476	16.9
Laterality	Left	874	31
	Right	869	30.8
	One side, NOS	7	0.2
	Bilateral, single primary	1002	35.6
	Paired site	66	2.3
Surgery	None	57	2
	Tumor removal	486	17.2
	Tumor removal with omentectomy	1056	37.5
	Cytoreductive surgery	1124	39.9
	Pelvic exenteration	40	1.4
	Unknown	55	2

### Epidemiological trend

**Fig. 1:**
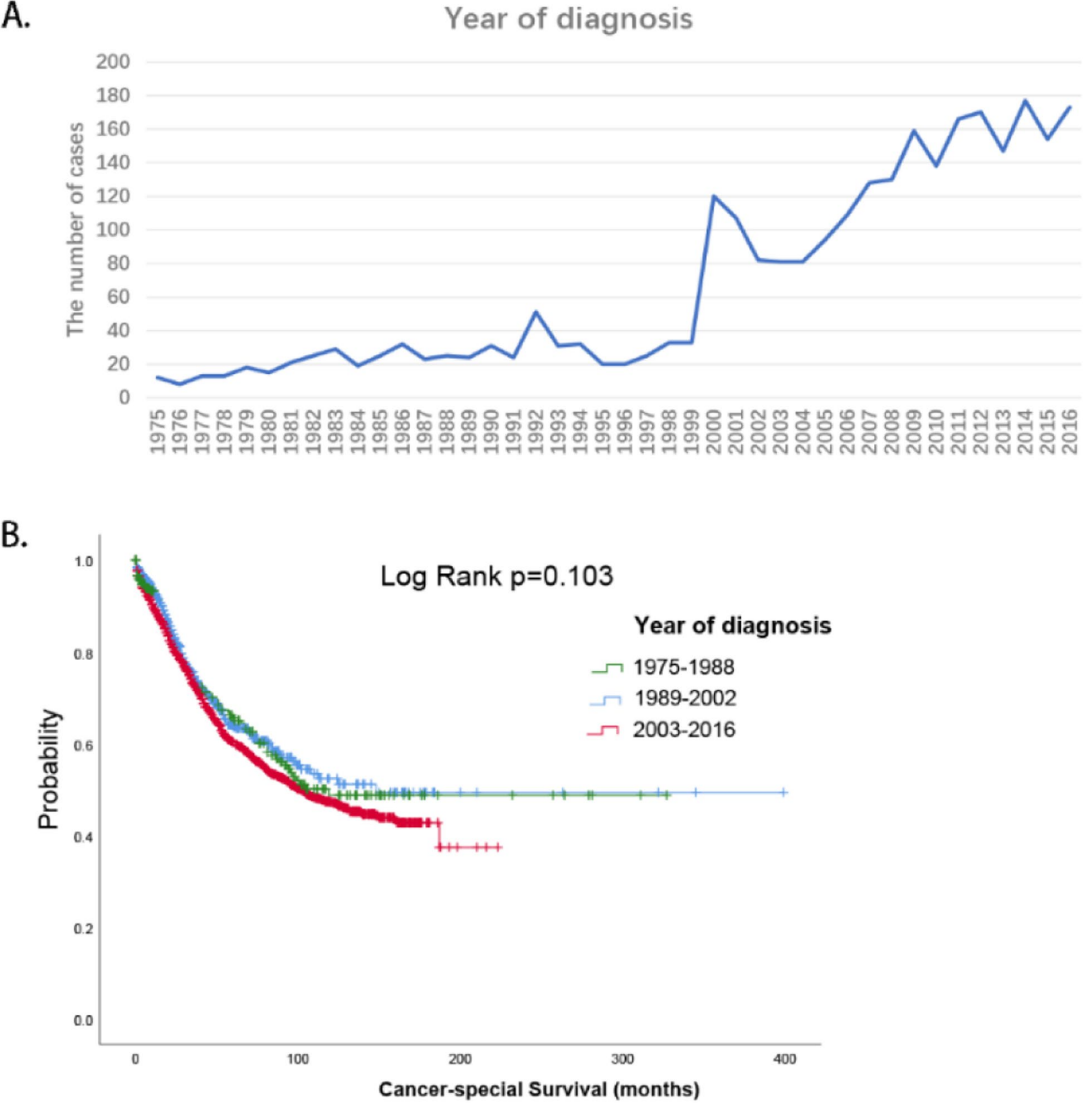
Epidemiological trend. **A** Annual distribution of the mixed cell adenocarcinoma patients registered in SEER. **B** Prognosis of mixed cell adenocarcinoma patients in different periods

The number of MCOA cases registered in the SEER database has been increasing for the past 40 years (Fig. 1A). Then, we divided MCOA patients enrolled in the SEER database from 1975 to 2016 into an average of three groups per year. Survival analysis showed that in 1975-1988, 1989-2002, and 2003-2016, the estimated median survival was 119 months, 148 months, and 102 months, respectively, and there was no significant difference (*p*=0.103, Fig. 1B).

### Identification of independent prognostic factors

Furthermore, the variables potentially influencing overall survival were analyzed using univariate Cox proportional hazards analysis. The results are shown in Table 2. Age, tumor size, grade, laterality, SEER stage, AJCC stage, surgical resection, and the number of malignant tumors in situ were significantly associated with the prognosis of mixed cell adenocarcinoma (*p*<0.05). Then, all these variables were incorporated into a further multivariate Cox regression analysis to identify independent prognostic factors. The results showed that age (HR=1.2848, *p*<0.0001), grade (HR=1.2594, *p*<0.0001), SEER stage (HR=1.6315, *p*=0.0002) and AJCC stage (HR=1.5921, *p*<0.0001) were independent prognostic factors for mixed cell adenocarcinoma.

**Table 2:** Univariate and multivariate analysis

Variables	Univariate analysis		Multivariate analysis	
	HR(95%CI)	*p*	HR(95%CI)	*p*
Race	1.0708(0.9457,1.2124)	0.28		
Age	1.459(1.3095,1.6255)	<0.0001	1.2848(1.1492,1.4364)	<0.0001
Size	0.7884(0.7044,0.8824)	<0.0001	0.894(0.7964,1.0035)	0.0574
Grade	1.6288(1.4705,1.8042)	<0.0001	1.2594(1.1212,1.4146)	<0.0001
SEER stage	3.5691(3.0598,4.1632)	<0.0001	1.6315(1.2518,2.1262)	0.0002
AJCC Stage	2.241(2.0562,2.4424)	<0.0001	1.5921(1.3617,1.8615)	<0.0001
Laterality	1.52(1.3744,1.681)	<0.0001	1.0563(0.953,1.1709)	0.2965
Surgery	1.7553(1.5656,1.9679)	<0.0001	1.0295(0.9109,1.1637)	0.6406

### Survival analysis

**Fig. 2:**
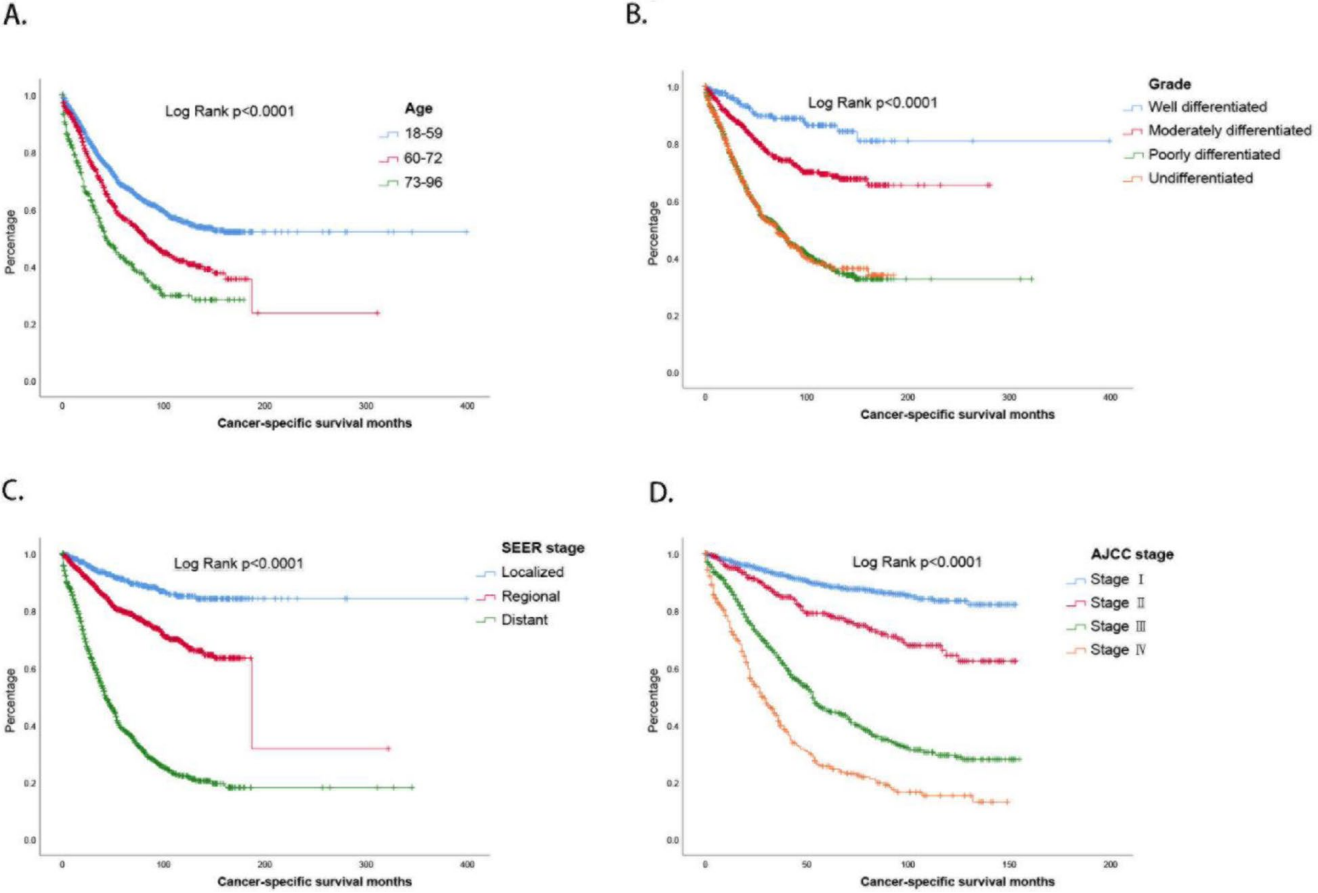
Survival analysis **A**. Age **B**. Grade **C**. SEER stage **D**. AJCC stage

Then, we performed a Kaplan–Meier survival analysis for prognostic independent factors of mixed cellular adenocarcinoma. The results show that the 5-year CSS (cancer-specific survival) rates for patients in the 18-59, 60-72, and 73-96 age groups were 68.1, 56.9, and 42.9%, respectively (Fig. 2A). Grouped according to the differentiation of tumor cells, poorly differentiated and undifferentiated patients had almost identical survival curves, with median survival times of 74 and 71 months, respectively. In addition, the 5-year survival rates for well-differentiated and moderately differentiated patients were 89.6 and 77.4%, respectively (Fig. 2B). SEER stages were associated with the invasion and metastasis of mixed cell adenocarcinoma, and the 5-year CSS rates of patients with localized, regional and distant disease were 91.1, 79.6, and 38.4%, respectively (Fig. 2C). The prognosis of patients with different AJCC stages was statistically significant, and the 5-year CSS rates of stage I, stage II, stage III, and stage IV were 88.8, 78.5, 45, and 25.6%, respectively (Fig. 2D).

### Construction and verification of CSS nomogram

**Fig. 3:**
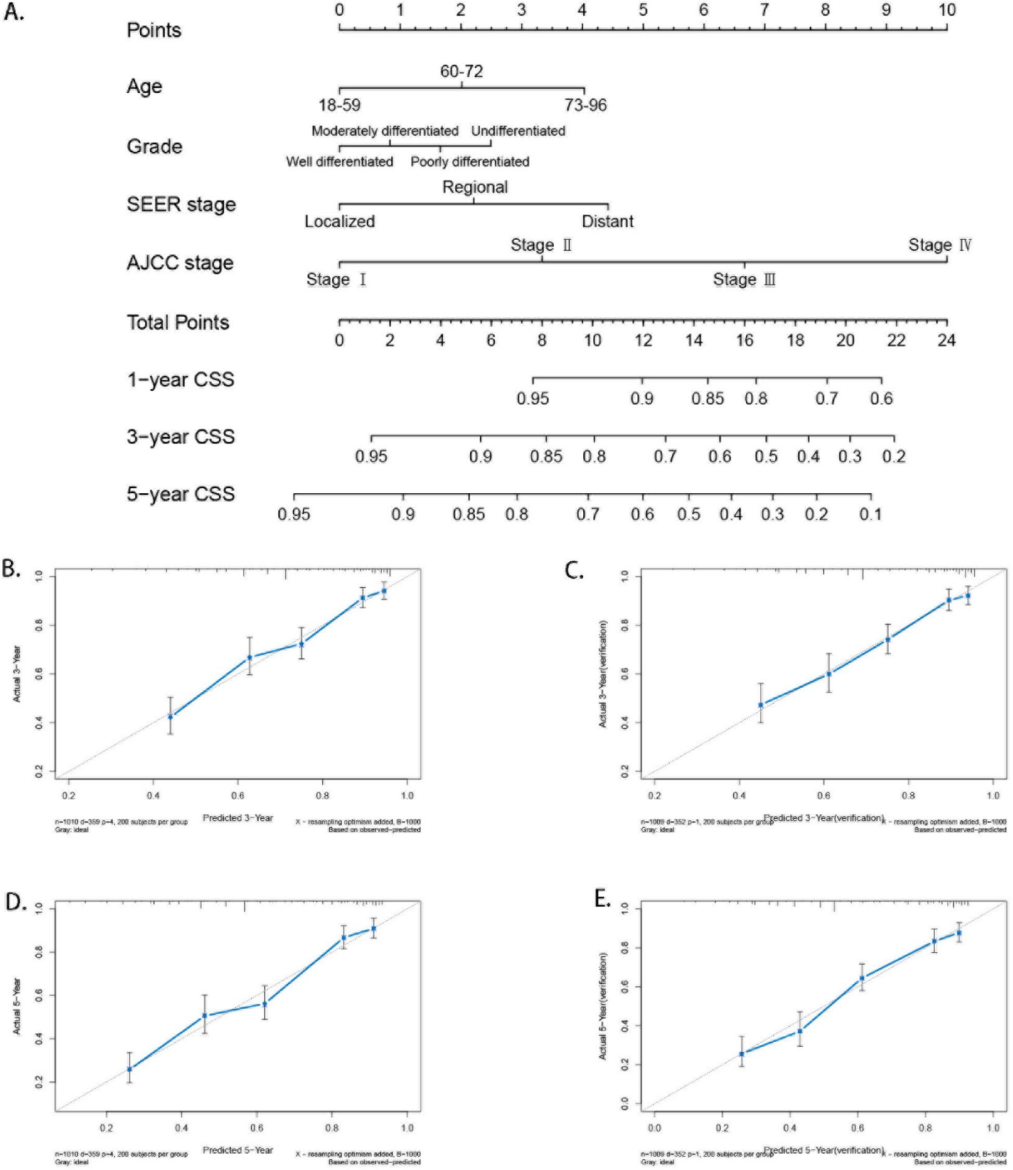
Nomogram and calibration plots **A**. Nomogram for mixed cell adenocarcinoma **B** and **C**. Calibration plots for internal validation of 3- and 5-year survival C and D. Calibration plots for external validation of 3- and 5-year survival

Subsequently, the four independent prognostic factors were used to construct a nomogram to predict patient survival. A total of 2,019 MCOA patients with complete clinical characteristics were randomly divided into the modeling group and the validation group. No significant difference in clinical characteristics was found between the modeling group and the validation group (Table 3). Finally, a nomogram was constructed from the data of 1010 mixed cell adenocarcinoma patients, with an internally verified C-index of 0.743 (Fig. 3A). In the validation group, 1009 patients were used for external validation, and the C-index was 0.731, which means that our nomograms have moderate predictive efficiency. The calibration diagram also shows consistency between the predicted and actual values (Fig. 2B, C, D, and E). This nomogram assigns scores to each variable, which are added and compared with the scale to predict patient survival time.

**Table 3:** Clinical characteristics of patients in the modeling and validation groups

Variable	Classification	Modeling group	Validation group	*p*
n		(1010)	(1009)	
Age (%)	18-59	563 (55.7)	598 (59.3)	0.268
	60-72	305 (30.2)	277 (27.5)	
	73-96	142 (14.1)	134 (13.3)	
Grade (%)	Well differentiated	86 ( 8.5)	97 ( 9.6)	0.556
	Moderately differentiated	192 (19.0)	189 (18.7)	
	Poorly differentiated	465 (46.0)	438 (43.4)	
	Undifferentiated	267 (26.4)	285 (28.2)	
AJCC stage (%)	Stage I	342 (33.9)	356 (35.3)	0.103
	Stage II	144 (14.3)	158 (15.7)	
	Stage III	373 (36.9)	381 (37.8)	
	Stage IV	151 (15.0)	114 (11.3)	
SEER stage (%)	Localized	180 (17.8)	209 (20.7)	0.229
	Regional	344 (34.1)	341 (33.8)	
	Distant	486 (48.1)	459 (45.5)

### Survival analysis before and after PSM

**Fig. 4:**
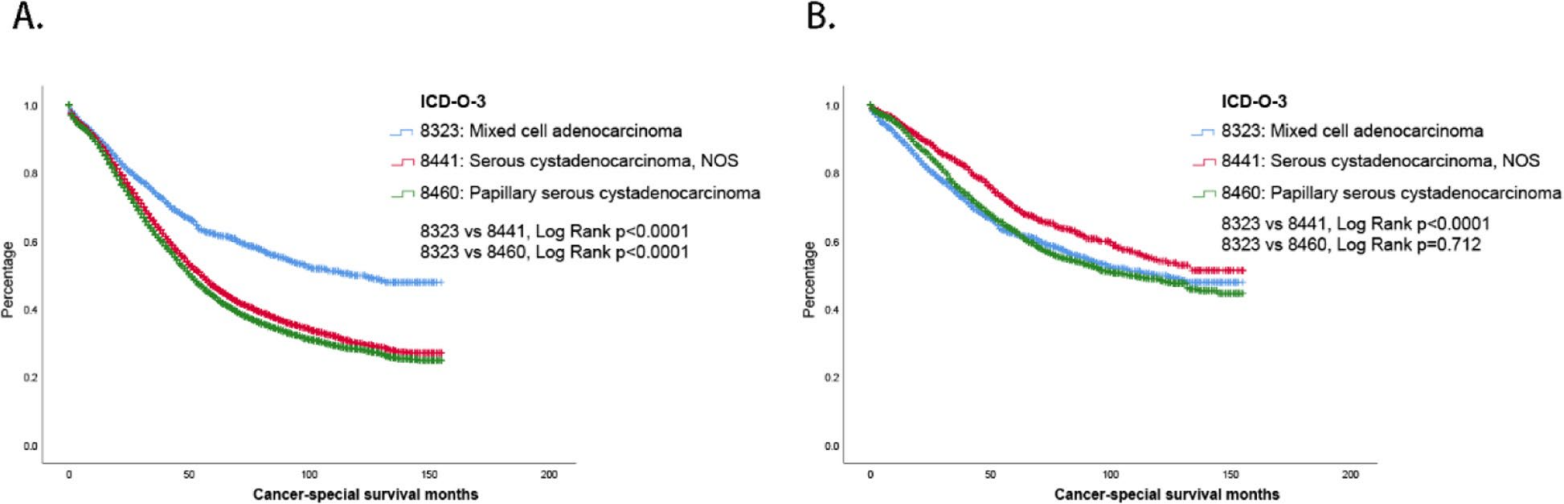
Survival analysis of mixed cell adenocarcinoma, serous cystadenocarcinoma, and papillary serous cystadenocarcinoma **A**. Before PSM **B**. After PSM

Serous cystadenocarcinoma and papillary serous cystadenocarcinoma are two common pathological types of ovarian carcinomas. In this study, we performed a survival analysis for ovarian mixed cell adenocarcinoma, serous cystadenocarcinoma, and papillary serous cystadenocarcinoma using data from the SEER database. The results showed that the 5-year survival rates of ovarian mixed cell adenocarcinoma, serous cystadenocarcinoma, and papillary serous cystadenocarcinoma were 62, 46.7, and 43.8%, respectively. Statistically, mixed cell adenocarcinoma had a significantly better prognosis than serous cystadenocarcinoma or papillary serous cystadenocarcinoma (Fig. 4A, *p*<0.0001). However, these conclusions are biased because confounding factors have not been calibrated. Significant differences in age, grade, SEER stage, and AJCC stage were found among the three carcinomas (Table 4). Therefore, we conducted PSM analysis with mixed cell adenocarcinoma as a reference and finally identified 2,019 serous cystadenocarcinomas and papillary serous cystadenocarcinomas. Survival analysis after PSM showed a 5-year survival rate of 69.7% for serous cystadenocarcinoma and 62.9% for papillary serous cystadenocarcinoma (Fig. 4B). These results mean that serous adenocarcinoma had the best prognosis of the three pathologic types of ovarian carcinoma (*p*<0.0001), with no significant difference between papillary serous cystadenocarcinoma and mixed cell adenocarcinoma (*p*=0.712). This is a dramatic difference from the results without PSM analysis.

**Table 4:** Clinical characteristics before and after PSM

Before PSM							After PSM			
		Mixed cell adenocarcinoma	Serous cystadenocarcinoma	*p*	Papillary serous cystadenocarcinoma	*p*	Serous cystadenocarcinoma	*p*	Papillary serous cystadenocarcinoma	*p*
n		2019	10790		7949		2019		2019	
Age	(mean (SD))	57.90 (12.42)	61.88 (12.07)	<0.001	61.52 (12.57)	<0.001	58.07 (12.37)	0.66	58.53 (12.60)	0.109
Grade (%)	Well differentiated	183 ( 9.1)	356 ( 3.3)	<0.001	365 ( 4.6)	<0.001	191 ( 9.5)	0.087	135 ( 6.7)	0.023
	Moderately differentiated	381 (18.9)	944 ( 8.7)		1313 (16.5)		322 (15.9)		359 (17.8)	
	Poorly differentiated	903 (44.7)	5163 (47.8)		4204 (52.9)		955 (47.3)		946 (46.9)	
	Undifferentiated	552 (27.3)	4327 (40.1)		2067 (26.0)		551 (27.3)		579 (28.7)	
SEER stage (%)	Localized	389 (19.3)	541 ( 5.0)	<0.001	354 ( 4.5)	<0.001	413 (20.5)	0.386	353 (17.5)	0.323
	Regional	685 (33.9)	1906 (17.7)		1245 (15.7)		647 (32.0)		710 (35.2)	
	Distant	945 (46.8)	8343 (77.3)		6350 (79.9)		959 (47.5)		956 (47.4)	
AJCC stage (%)	Stage I	698 (34.6)	1010 ( 9.4)	<0.001	706 ( 8.9)	<0.001	699 (34.6)	0.99	671 (33.2)	0.631
	Stage II	302 (15.0)	945 ( 8.8)		627 ( 7.9)		297 (14.7)		329 (16.3)	
	Stage III	754 (37.3)	5954 (55.2)		4481 (56.4)		762 (37.7)		758 (37.5)	
	Stage IV	265 (13.1)	2881 (26.7)		2135 (26.9)		261 (12.9)		261 (12.9)	

## Discussion

Most women with ovarian cancer are diagnosed at an advanced stage. Approximately 70 to 80% of these women have relapses, and 75% have no cure [10]. Ovarian cancer is the gynecological malignancy with the highest mortality rate. Studies have shown that death rates from ovarian cancer are falling in Western countries, but this trend is more likely to be related to increased use of contraceptives and a decline in postmenopausal hormone replacement therapy than to better treatments [11]. Surgery is a potential treatment for the complete removal of tumors. However, the choice between laparotomy and minimally invasive surgery for ovarian cancer is currently controversial. ESGO-ESMO guidelines still recommend laparotomy as the standard method for patients with early-stage ovarian cancer [12]. Interestingly, a recently published multicenter observational retrospective study showed that appropriately selected patients with early-stage ovarian cancer could benefit from minimally invasive surgery [13].

SOC is the most common tissue type of epithelial ovarian cancer. SOC is < 5% LGSOC [7] and mostly HGSOC. LGSOC accounts for 6-8% of ovarian cancers [14]. Lymph node metastasis is an independent factor of the poor prognosis of LGSOC [15]. Although LGSOC is not sensitive to chemotherapy drugs, its prognosis is still better than that of HGSOC [16]. In addition, mucinous ovarian adenocarcinoma accounts for 10% of all ovarian cancers and 4% of advanced ovarian cancers, mostly at stage I, with a good prognosis at an early stage [7]. Studies have described that women with serous papillary ovarian cancer have a significantly worse prognosis than women with stage III-IV serous papillary ovarian cancer [17, 18]. However, clinically, only single histological ovarian cancer has been reported in most cases, and MCOA of multiple histological types has been reported only as individual cases, which limits our understanding of the clinicopathological and prognostic characteristics of this disease. In our study, we described the clinicopathologic features of 2,818 patients with MCOA from 1975 to 2016 from the SEER database and demonstrated factors affecting CSS. In addition, we developed a prognostic nomogram with moderate efficacy to visually predict 1-, 5-, and 10-year survival to guide clinical decision-making. Epidemiologically, we found an increasing trend in the number of MCOA patients in the SEER database. Moreover, there was no statistically significant difference in survival between the three periods. Therefore, further study of MCOA is of great value.

In this study, we found MCOA with a median age of 57 and a median tumor size of 110 mm. Most patients are diagnosed with clinically advanced disease, but metastasis is rare. In addition, the results suggest that prognosis does not differ between human races, which is inconsistent with the racial differences shown in the incidence of all ovarian cancers from the database [19–22]. Notably, age, grade, SEER stage, and AJCC stage were potential independent risk factors for MCOA in the univariate and multivariate analyses. It was found that the older the age was, the lower the overall survival rate. In addition to acting as an independent prognostic factor, pathological grade can also be a determinant of treatment.

In terms of treatment, surgery and chemotherapy are the main treatments to eliminate as many cancer cells as possible. Of course, some scholars have proposed that the probability of achieving cancer-free status is maximized by combining tumor cell reduction surgery and intraperitoneal drug chemotherapy. Up to 50% of women with advanced ovarian cancer can be cured with this method [8]. In our clinical practice, the early stage of the disease is still dominated by surgery. The surgical methods are total hysterectomy, bilateral fallopian tube, ovarian resection, and comprehensive stage exploration to completely remove the tumor and avoid tumor rupture as much as possible [23]. Young patients who wish to retain fertility can undergo a unilateral fallopian tube and oophorectomy for stage I and low-risk ovarian tumors (early stage, low-grade aggressive tumors, and low malignant potential ovarian tumors). In the late or infiltrating phase, cytoreductive surgery is the main method and is also the initial treatment recommendation for patients with stages II, III, and IV disease. Although it is a standard treatment, it is a recommendation based on retrospective research data [24]. The benefits of cytoreductive surgery after neoadjuvant therapy are still controversial. Patients with stage III/IV large tumors who are not suitable for surgery should be considered, and the pathological diagnosis should be clear before initiating neoadjuvant therapy.

Finally, we established a nomogram to predict the prognosis of MCOA patients, with moderate repeatability and reliability. In addition, we compared the prognosis of ovarian cancer patients with different pathologic types. SOC and serous papillary adenocarcinoma are relatively more common tissue types in ovarian cancer. Through PSM analysis, we corrected the confounding bias caused by age, rating, SEER stage, and AJCC stage and identified candidate patients. MCOA had the worst prognosis, and SOC had the best prognosis in the three groups. This finding gives us a better understanding of rare MCOA patients.

Inextricably, our results also have certain limitations. For example, some patients lack data on key variables, which may lead to biased results. To retain a sufficient sample size, we included patients who lacked a few variables in the analysis. We do not know of treatment methods other than surgery, which could confound the results. We cannot estimate the potential impact of these treatments on prognosis, thus limiting our ability to describe the impact of other treatment methods on prognosis. Finally, the association between the occurrence of MCOA and genomic changes in patients remains unclear.

## Conclusions

In summary, MCOA is uncommon, and age, grade, SEER stage, and AJCC stage are independent prognostic factors. Compared with other common malignant ovarian tumors, MCOA has a poor prognosis. A nomogram was constructed to predict survival time and assist in clinical decision-making.

## Data Availability

The dataset(s) supporting the conclusions of this article is (are) included within the article
